# TOCA-2 regulates gonad development in *C. elegans*

**DOI:** 10.17912/micropub.biology.002264

**Published:** 2026-08-04

**Authors:** Yogesh Pratap, Tanushree Sinha, Anup Padmanabhan

**Affiliations:** 1 Department of Biology, Trivedi School of Biosciences, Ashoka University, Sonipat, Haryana, India; 2 Max Planck School Matter to Life, Heidelberg, BW, Germany

## Abstract

*C. elegans* gonad development and maintenance involve the coordinated integration of biochemical signalling and mechanical forces. Here, we identify a previously unrecognized role for *Ce* TOCA-2 , the *C. elegans* ortholog of mammalian TOCA-1 , in maintaining the structural integrity and morphogenesis of the *C. elegans* gonad. Animals lacking TOCA-2 exhibit pronounced architectural defects, including aberrant gonad morphology, premature distal gonad shrinkage, and disorganized syncytial germline, which alter the spatial patterns of cytoplasmic flow in the syncytium. Our findings establish TOCA-2 as a key regulator of gonad morphogenesis and an important link between cytoskeletal organization and organogenesis in *C. elegans* .

**Figure 1. TOCA-2 depletion leads to loss of germline architecture and defective gonad morphology:** **A)** Representative image of adult *C. elegans* gonad morphology in control and *toca-2 (null)* animals. Top: Epifluorescence image of a control worm showing the gonad (green) and intestine (red). The two ‘U-shaped' gonadal arms are bilaterally symmetric mirror images of each other, with one arm passing above the intestine and the other below the intestine. Schematic showing regions of *C. elegans* gonad: (1) Distal tip cell, (2) progenitor (mitotic cell cycle) zone, (3) Pachytene, (4) Diplotene, (5) Diakinesis, (6) Spermatheca, (7) Embryo within the uterus. Bottom: Images of *toca-2 (null)* gonads showing various morphological defects. Dotted yellow lines represent the curvature of the gonadal arm. Scale bar: 20µm. **B)** Plot showing the percentage of gonad morphological defects in control, *toca-2 (null)* , *toca-2 (RNAi)* , *toca-2 (null); toca-1 (null),* and *toca-2 (null* ); *P ^pie-1^* :: TOCA-2 ::GFP animals. C) Quantification of gonad morphology defect types in control and *toca-2 (RNAi)* animals. Bars represent the percentage of animals displaying different types of defects. **D)** Morphometric quantification of gonad compression and directional defect. Schematic details the measurements used to quantify compression (R _w_ ) and directional displacement (R _D_ ), in control and *toca-2 (RNAi)* . Each data point represents the gonad of a single animal. **E)** Images depicting gonad development over time in control and *toca-2 (null)* animals. A red-filled arrowhead indicates the defective gonad in later stages of development in *toca-2 (null)* worms. Scale bar: 50 µm. **F)** Top: Scatter plot showing quantification of gonad length in control and *toca-2 (null)* worms at 24-hour intervals (n=16). Bottom: The line graph of the scatter plot shown above. Error bars indicate standard deviation. **G)** Quantification of embryo size in control and *toca-2 (null)* worms. **H)** Confocal images indicating the progenitor zone (red line) in the gonads of control (n=6) and *toca-2 (null)* (n=12) animals expressing mCherry:: HIS-58 . Scale bar, 50 µm. **I)** Confocal images of Phalloidin ^647^ stained distal region of syncytium from control and *toca-2 (null)* animals. Scale bar: 50 µm. **J)** Particle Image Velocimetry (PIV) analysis of the cytoplasmic flow in control and *toca-2 (null)* syncytium. Scale bar: 10 µm. **K)** Averaged instantaneous cytoplasmic streaming velocities of all spatial interrogation windows in consecutive frames in control (n=4) and *toca-2 (null)* (n=6) syncytium. **L)** Contour plots of time-averaged instantaneous velocities across all 210 interrogation windows in the distal gonad arm of control and *toca-2 (null)* animals. PIV was performed using an interrogation window of 32x32 pixels with 50% overlap over an ROI of 282×264 pixels (30.32×28.38 µm). Statistical significance was determined using the Mann-Whitney U test. ****p<0.0001, ***p <0.001, **p <0.01 and * p <0.05.

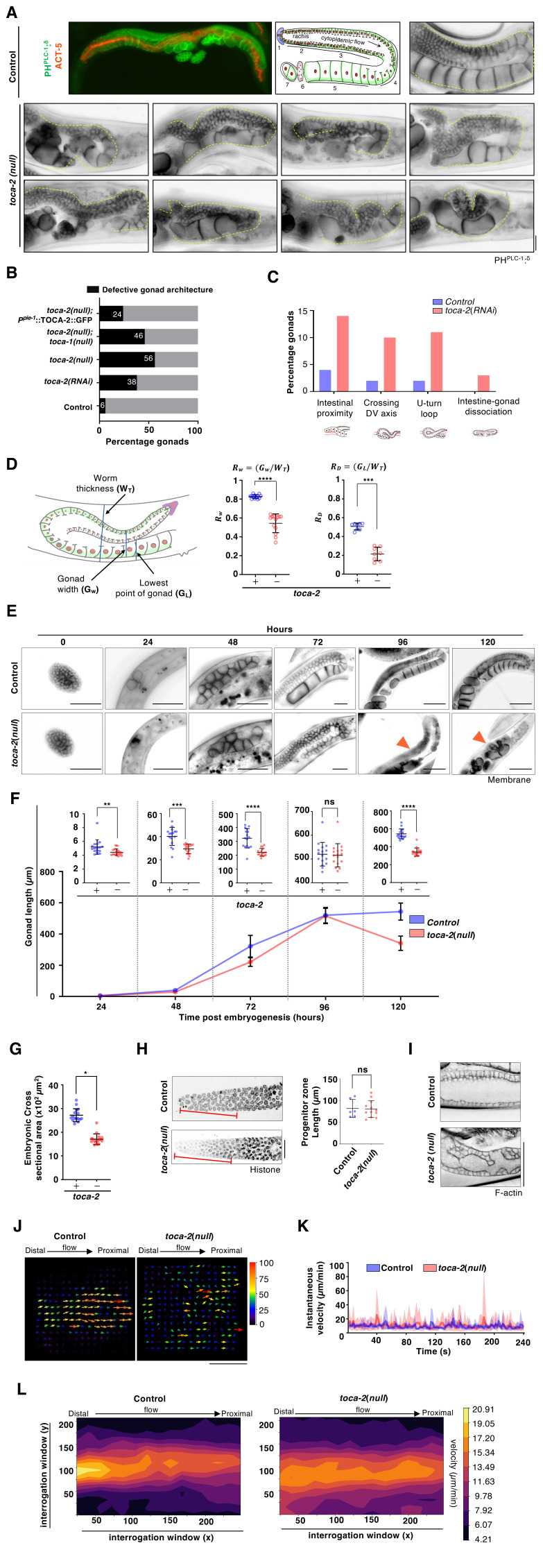



## Description

The *C. elegans* hermaphrodite gonad comprises two symmetrically arranged U-shaped tubes, with one gonad arm positioned above the intestine and the other below it, giving rise to a characteristic ‘hugging' morphology (Hubbard & Greenstein, 2000; McGhee, 2007; Pazdernik & Schedl, 2013). Post-embryonic development of the *C. elegans* hermaphrodite gonad involves germline proliferation, collective cell migration, differentiation and basement membrane remodelling-processes that require coordinated regulation of cytoskeletal architecture and dynamic reorganization of the extracellular matrix (Agarwal et al., 2022). The TOCA family of proteins regulate membrane-cytoskeleton interactions through specialized functional domains. *C. elegans* expresses two TOCA-2 paralogs, *Ce* TOCA-1 and *Ce* TOCA-2 , that form an autosome/X gene pair (Maciejowski et al., 2005). Germline specific expression of *Ce* TOCA-1 and *Ce* TOCA-2 , resulted in their localization to the germline, rachis membranes and early embryos (Giuliani et al., 2009; Nageswaran et al., 2025). Domain analysis revealed that both proteins contain an N-terminal F-BAR domain involved in membrane curvature sensing, an HR1 domain that binds CDC-42 , and a C-terminal SH3 domain that interacts with N-WASP. Previous studies have shown that depletion of *Ce* TOCA-2 (hereafter TOCA-2 ) disrupts clathrin-mediated endocytosis of intestinal yolk uptake into the germline, impairing oocyte maturation and reducing brood size (Giuliani et al., 2009). Furthermore, the genetic null mutant allele, *toca-2 ( ng11 )* (hereafter *toca-2 (null)* ) exhibits increased embryonic lethality due to the *gex* ( *g* ut on the *ex* terior) phenotype (Giuliani et al., 2009; Soto et al., 2002).

To investigate the role of TOCA-2 in gonad morphogenesis and oocyte development, we examined *toca-2 (RNAi)* and *toca-2 (null)* animals co-expressing the intestinal marker ACT-5 ::mCherry and the germline membrane marker GFP::PH ^PLC-1 δ^ . Microscopic analysis revealed that depletion of TOCA-2 caused pronounced defects in gonad morphology and architecture ( Fig. 1A ). Whereas only 6% in control animals displayed abnormal gonad morphology, 36% in *toca-2 (RNAi)* and 56% in *toca-2 (null)* animals had defective gonad architecture ( Fig. 1A and 1B). Intriguingly, simultaneous depletion of TOCA-1 and TOCA-2 resulted in 46% of animals displaying gonad defects, suggesting that TOCA-2 , rather than TOCA-1 , plays a major role in gonad morphogenesis ( Fig. 1B ). This agrees with the fact that being an autosome/X pair, TOCA-1 is most likely silenced in the germline. Germline-specific expression of GFP- TOCA-2 in *toca-2 (null)* animals partially rescued the phenotype, reducing the frequency of defective gonads from 56% in *toca-2 (-/-)* animals to 24%. To further characterize the observed defects, we classified gonad abnormalities into four major categories; (1) failure of the gonad arms to properly navigate around the intestine, resulting in compression within a restricted region (Intestinal proximity); (2) loss of directional migration in the dorsal arm, leading to aberrant intersections along the dorsoventral (DV) axis (Crossing DV axis); (3) distortion of the characteristic ventral-to-dorsal U-turn (U-turn loop); and, (4) complete disassociation of the gonad from the intestine, disrupting the normal “hugging' morphology (Intestine-gonad dissociation) ( Fig. 1C ). To quantitively assess the gonad compression, we measured two parameters: normalized gonad width (R _W_ ), and normalized dorsal edge displacement from the body wall (R _D_ ). TOCA-2 depletion resulted in ~50% reduction in R _W_ and R _D_ , confirming increased gonad compression (Fig.1D).

To investigate the temporal progression of these defects, we monitored gonad development in control, and *toca-2 (null)* animals expressing GFP::PH ^PLC-1 δ^ and mCherry:: HIS-58 in the germline at 24-hour intervals throughout their post-embryonic development. Consistent with previous reports, TOCA-2 depletion resulted in reduced brood size and significantly smaller embryos ( Fig. 1E and 1F)(Giuliani et al., 2009). Throughout development, *toca-2 (null)* animals exhibited delayed gonad expansion compared with controls. Interestingly, between 96-120 hours, mutant gonads exhibited pronounced structural defects accompanied by a significant reduction in gonad length compared to controls ( Fig. 1F ). This shrinkage was largely confined to the distal gonad arm ( Fig. 1E, red arrows). The phenotype resembled previously described age-associated gonad atrophy, although it occurred substantially earlier in *toca-2 (null)* animals (Day 4/ 96 hrs) than in wild-type animals (~Day 10 ) (De La Guardia et al., 2016). To determine whether altered germ cell proliferation contributed to impaired gonad elongation, we measured the length of the progenitor zone. However, no significant differences were detected between control and *toca-2 (null)* animals ( Fig. 1H ), suggesting that impaired germline proliferation is unlikely to account for the reduced gonad length. Similar germline proliferation between control and mutant animals also ruled out excessive germ cell accumulation and subsequent mechanical crumpling as the cause of gonad shrinkage.

TOCA-2 localizes to the partially ingressed rachis membranes and has been implicated in regulating actin dynamics and syncytial organization (Giuliani et al., 2009; Soto et al., 2002). We therefore hypothesized that loss of TOCA-2 disrupts the actomyosin corset surrounding the germline syncytium, thereby altering cytoplasmic flow and compromising tissue integrity. Whole-worm phalloidin staining revealed irregular syncytial morphology in *toca-2 (null)* animals ( Fig. 1I ). Consistent with this observation, Particle Image Velocimetry (PIV) analysis demonstrated altered cytoplasmic flow patterns in *toca-2 (null)* mutant gonads ( Fig. 1J ). Although the mean instantaneous velocities were comparable between control and mutant animals, the spatial velocity distribution showed significantly greater variability in *toca-2 (null)* gonads (standard deviation ~8 µm/s in *toca-2 (null)* versus ~5 µm/s in controls) ( Fig. 1K ). Time-average velocity maps further confirmed this altered flow organization: mutant gonads displayed elevated velocities near the syncytial boundaries, whereas control gonads exhibited smooth decline in velocity from the centre toward the periphery ( Fig. 1L ). Together these findings suggest that loss of TOCA-2 disrupts actomyosin organization within the germline syncytium, leading to aberrant cytoplasmic flow and defective gonad architecture.

Taken together, our results demonstrate that TOCA-2 is essential for maintaining both the overall morphology of the gonad and internal architecture of the germline syncytium. Germline-specific expression of TOCA-2 is sufficient to substantially rescue morphological defects, indicating a cell-autonomous role in gonad morphogenesis. Beyond its previously established function in clathrin-mediated yolk endocytosis, our findings identify TOCA-2 as a critical regulator of gonad organogenesis and tissue-scale mechanical homeostasis in the *C. elegans* germline.

## Methods

**Growth and maintenance of strains**

*C. elegans* and bacterial strains used in this study are listed in Table S1. Primers used to confirm deletion mutants in *toca-1* and *toca-2* are listed in Table S2. All *C. elegans* strains were maintained at 20° C on Nematode Growth Medium (NGM) agar plates seeded with *E. coli* OP50 (Stiernagle, 2006). All bacterial cultures were grown in Luria-Bertani (LB) broth at 37°C at 180 rpm.

**RNA interference**

RNA interference was performed as previously described (Kamath, 2003), by feeding of the *E. coli* strain HT115 (DE3) expressing the L4440 plasmid containing *toca-2* targeting sequence. HT115 (DE3) containing RNAi clones was cultured in LB broth containing ampicillin (100 μg/mL) and tetracycline (12.5 μg/mL) at 37° C and seeded on to the NGM plate containing 1mM isopropyl β-D-thiogalactoside (IPTG) and 100 μg/mL ampicillin as described previously (Kamath, 2003). HT115 (DE3) expressingL4440 (vector alone) was used as the RNAi control. F2 embryos from animals grown on *toca-2 (RNAi)* plates were isolated and allowed to hatch on plates devoid of bacteria. L1-stage synchronized worms were subsequently transferred to *toca-2 (RNAi)* plates for analyzing post embryonic development of germline architecture.

**Microscopy**

Gonad architecture was imaged using an Olympus BX63 Upright epi-fluorescence microscope. A total of 100 worms were examined from both the mutant and control groups ( N2 ). The worms were mounted on a 3% agarose pad and anesthetized with 0.05% levamisole. Images were acquired using Olympus CellSens Dimension software (version 2.3). Gonad showing significant morphological deviations from wildtype were counted as defective and the proportion of defective animals was compared across conditions using GraphPad prism version 10.6.1.

**Mitotic Zone Analysis**

Worms were washed in M9 buffer, anesthetized in 0.025% levamisole and were dissected near the pharynx using a hypodermic needle. Extruded gonads were fixed in 2% PFA (Paraformaldehyde) for 15 minutes and imaged on an Olympus IX83 Inverted Microscope (Spinning-disc confocal) and excited at 488 nm and 561 nm laser lines using an OBIS Coherent laser system. The length of the progenitor (mitotic cell cycle) zone on the distal side of the gonad was measured from the distal tip cell (DTC) to the transition zone, characterized by two or more crescent-shaped nuclei in a row.

**Particle Image Velocimetry (PIV)**

PIV for cytoplasmic flow in distal arm of the gonad was carried out through an ImageJ plugin. Time-lapse DIC videos were recorded for 4 minutes at 2-second intervals (120 frame pairs). PIV was performed on an ROI of size 282×264 pixels (30.32×28.38 µm) using an interrogation window size of 32×32 pixels with 50% overlap, yielding 210 interrogation windows (edge pixels were excluded). Time-averaged velocity was calculated by averaging instantaneous velocities of a single interrogation window across all 120 frame pairs and spatial velocity was calculated by averaging instantaneous velocities across all interrogation windows for a single frame pair.

**Statistical Analysis**

The Mann-Whitney test and Student's t-test was employed in case of non-normal and normal distributed data, respectively.

## Reagents

Table S1: List of *C. elegans* strains

**Table:** 

**S. No.**	**Strain Name**	**Genotype**	**Source**
1	GU1145	*unc-119 ( ed3 )* III; *toca-2 ( ng11 )* III; *pwIs830[ pie-1 :: toca-2 ::GFP] *	Scita Lab / Barth Lab
2	APN031	u *nc-119 ( ed3* )III; *ltIs37* IV; *ltIs38* + *jyIs17 [vha-6p::mCherry:: act-5 ] *	Lab stock
3	GU1165	*toca-2 ( ng11 )* III; *toca-1 ( tm3334 )* X	Alex Hajnal Lab
4	OD95	*ltIs37* *[ pie-1 p::mCherry:: his-58 ]* *unc-119* (+)] IV; *ltIs38 [ pie-1 p::GFP::PH(PLC1δ) + unc-119 (+)* ]	CGC
5	APN035	*toca-2 ( ng11 )* III; *ltIs37* *[ pie-1 p::mCherry:: his-58 ]* + *unc-119* (+)] IV. *ltIs38 [ pie-1 p::GFP::PH(PLC1δ) + unc-119 (+)* ]	This study ( GU1165 X OD95 )
6	APN036	*toca-1 ( tm3334* )X; *ltIs37* *[ pie-1 p::mCherry:: his-58 ]* + *unc-119* (+)] IV. *ltIs38 [ pie-1 p::GFP::PH(PLC1δ) + unc-119 (+)* ]	This study ( GU1165 X OD95 )
7	APN018	*jyIs17 [vha-6p::mCherry:: act-5 ] *	Lab stock

Table S2: Primer Sequences used for cloning RNAi feeding constructs

**Table:** 

*toca-2* forward primer (RZB 276)	GCCACTCGACATCAAGTATAAGAATTC
*toca-2* reverse primer (APO 275)	GATGCGTAAATCGACACATAGCGGTG
*toca-1* forward primer (APO 358)	CGAGCCAGCATCGAGTTGGAG
*toca-1* reverse primer (APO 359)	TCTGATTAACACAAGACTCGGCCTC
